# Supplementary material for: Pan-cancer characterization of m6A-mediated regulation of T cell exhaustion dynamics and clinical relevancies in human cancers
Source: Mol Ther Nucleic Acids. 2025 Jan 25;36(1):102465. doi: 10.1016/j.omtn.2025.102465 (PMC11847731; doi:10.1016/j.omtn.2025.102465)
Supplement: Document S1. Figures S1 and S2 [file mmc1.pdf]

OMTN, Volume 36

## Supplemental information

# Pan-cancer characterization of m6A-mediated regulation of T cell exhaustion dynamics and clinical relevancies in human cancers

**Weiping Ji, Ye Fang, Liwei Chen, Yitong Zheng, Yifei Pei, Changqiu Mei, and Meng Zhou**

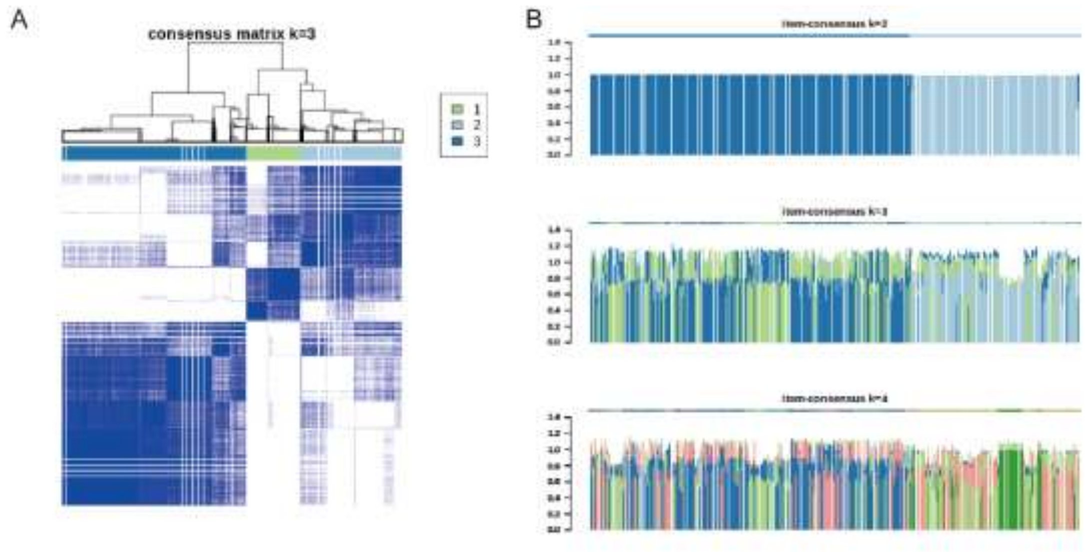


**Figure S1. Consensus clustering.** (A) Consensus matrix heatmap of the chosen optimal cluster number (k =3). (B) Item consensus for different cluster number

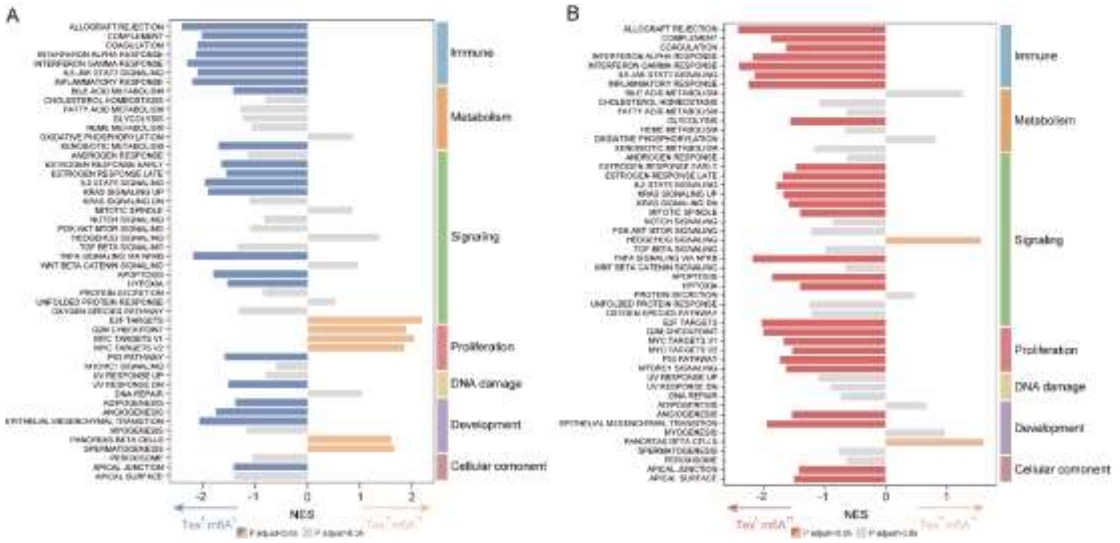


**Figure S2.** Enrichment analysis for the MSigDB hallmark pathways between TexLm6A$^{L}$ and TexHm6A$^{H}$ (A), and between Tex$^{L}$m6A$^{H}$ and Tex$^{H}$m6A$^{H}$ (B).

**Supplemental Tables**

**Table S1.** List of 518 T-cell exhaustion related genes and 31 m6A regulators.

**Table S2.** List of differentially expressed m6A regulators between normal T cells and exhausted T cells,

**Table S3.** List of differentially expressed m6A regulators between cytotoxic and exhausted T cells.
