# Supplementary material for: Pan-cancer characterization of m6A-mediated regulation of T cell exhaustion dynamics and clinical relevancies in human cancers
Source: Mol Ther Nucleic Acids. 2025 Jan 25;36(1):102465. doi: 10.1016/j.omtn.2025.102465 (PMC11847731; doi:10.1016/j.omtn.2025.102465)
Supplement: Document S2. Article plus supplemental information [file mmc3.pdf]

Molecular Therapy
Nucleic Acids
Original Article

Pan-cancer characterization of m6A-mediated regulation of T cell exhaustion dynamics and clinical relevancies in human cancers

Weiping Ji,[1,2] Ye Fang,[1,2] Liwei Chen,[1] Yitong Zheng,[1] Yifei Pei,[1] Changqiu Mei,[1] and Meng Zhou[1]

[1]Department of Genaral Surgery, School of Biomedical Engineering, The Quzhou Affiliated Hospital of Wenzhou Medical University, Quzhou People's Hospital, Wenzhou Medical University, Zhejiang, P.R. China

T cell exhaustion (TEX) is a major barrier to effective immunotherapy. The role of N6-methyladenosine (m6A) modification in regulating immune cell function has been recognized, but its impact on TEX dynamics across cancer types and clinical outcomes remains unclear. Here, we conducted a pan-cancer analysis integrating multi-omics data from cell lines, single-cell RNA sequencing, and pan-cancer and immunotherapy datasets to explore the dynamic interplay between m6A modification and TEX. We found that m6A modification influences key TEX-associated genes at both the cellular and single-cell levels, with distinct expression patterns across the exhaustion spectrum. Based on m6A-TEX interactions, three pan-cancer subtypes were identified, each with unique molecular profiles, immune phenotypes, and survival outcomes. The $Tex^{L}m6A^{L}$ subtype, characterized by low m6A activity and low TEX, correlated with high immune infiltration, increased cytolytic activity, and favorable survival, whereas the $Tex^{L}m6A^{H}$ and $Tex^{H}m6A^{H}$ subtypes with higher m6A activity were associated with poorer survival. Multivariate analysis confirmed the prognostic value of this classification independent of traditional clinical factors. Moreover, m6A-TEX crosstalk influenced responses to immune checkpoint blockade therapies. Our findings provide novel insights into the role of m6A in TEX regulation and underscore the potential of m6A regulators as biomarkers and therapeutic targets for advancing cancer immunotherapy.

INTRODUCTION

T cell exhaustion (TEX) is a distinct state of T cell dysfunction that occurs in chronic infections and cancer.[1] Exhausted T cells have a unique functional, phenotypic, and molecular profile that distinguishes them from naive, effector, and memory T cells; they are characterized by the progressive loss of effector functions, including reduced cytokine production, impaired proliferative capacity, and increased expression of immune checkpoint receptors such as programmed cell death 1 (PD-1).[2,3] TEX has emerged as a major barrier limiting the efficacy of immunotherapies.[4] The development of TEX is progressive and heterogeneous, occurring in a dynamic, hierarchical manner involving distinct subpopulations of exhausted T cells with distinct phenotypic and mechanistic characteristics within the tumor microenvironment (TME).[5,6] These subpopulations evolve along a continuum, influenced by complex transcriptional and epigenetic programs that ultimately shape the immune response against cancer.[6–11] Therefore, elucidating the molecular mechanisms and regulation underlying TEX dynamics is crucial for improving immunotherapy by redirecting T cells away from a dysfunctional developmental trajectory.

N6-methyladenosine (m6A) modification is one of the most important and dynamic regulators of gene expression.[12] m6A modification regulates a wide range of cellular processes, including RNA stability, splicing, translation, and decay, and plays an essential role in cancer biology.[13,14] Recent studies have highlighted the involvement of m6A in immune regulation, influencing the differentiation, activation, and function of various immune cells, including T cells.[15,16] In addition, m6A has also been implicated in modulating the tumor immune microenvironment, contributing to immune evasion mechanisms that allow tumors to evade immune surveillance.[17–19] Specifically, m6A may regulate T cell differentiation and fate decisions, influencing TEX and rejuvenation.[20] However, the comprehensive landscape of m6A regulator-mediated TEX dynamics across different cancer types and its clinical implications remain poorly understood.

In this study, we aimed to investigate the role of m6A modification in TEX across multiple cancer types by focusing on the crosstalk between m6A regulators and key TEX-related genes, and exploring how this interaction influences TEX, survival outcomes, and responses to immunotherapy.

RESULTS

m6A modulation of TEX dynamics at the cellular level

We compiled a catalog of 31 m6A genes that function as significant regulators of m6A-epitranscriptomic mechanisms. This catalog

Received 23 November 2024; accepted 22 January 2025;
https://doi.org/10.1016/j.omtn.2025.102465.

[2]These authors contributed equally

Correspondence: Meng Zhou, Department of Genaral Surgery, School of Biomedical Engineering, The Quzhou Affiliated Hospital of Wenzhou Medical University, Quzhou People's Hospital, Wenzhou Medical University, Zhejiang, P.R. China.
E-mail: zhoumeng@wmu.edu.cn

Published by Elsevier Inc. on behalf of The American Society of Gene and Cell Therapy.
This is an open access article under the CC BY license (http://creativecommons.org/licenses/by/4.0/).

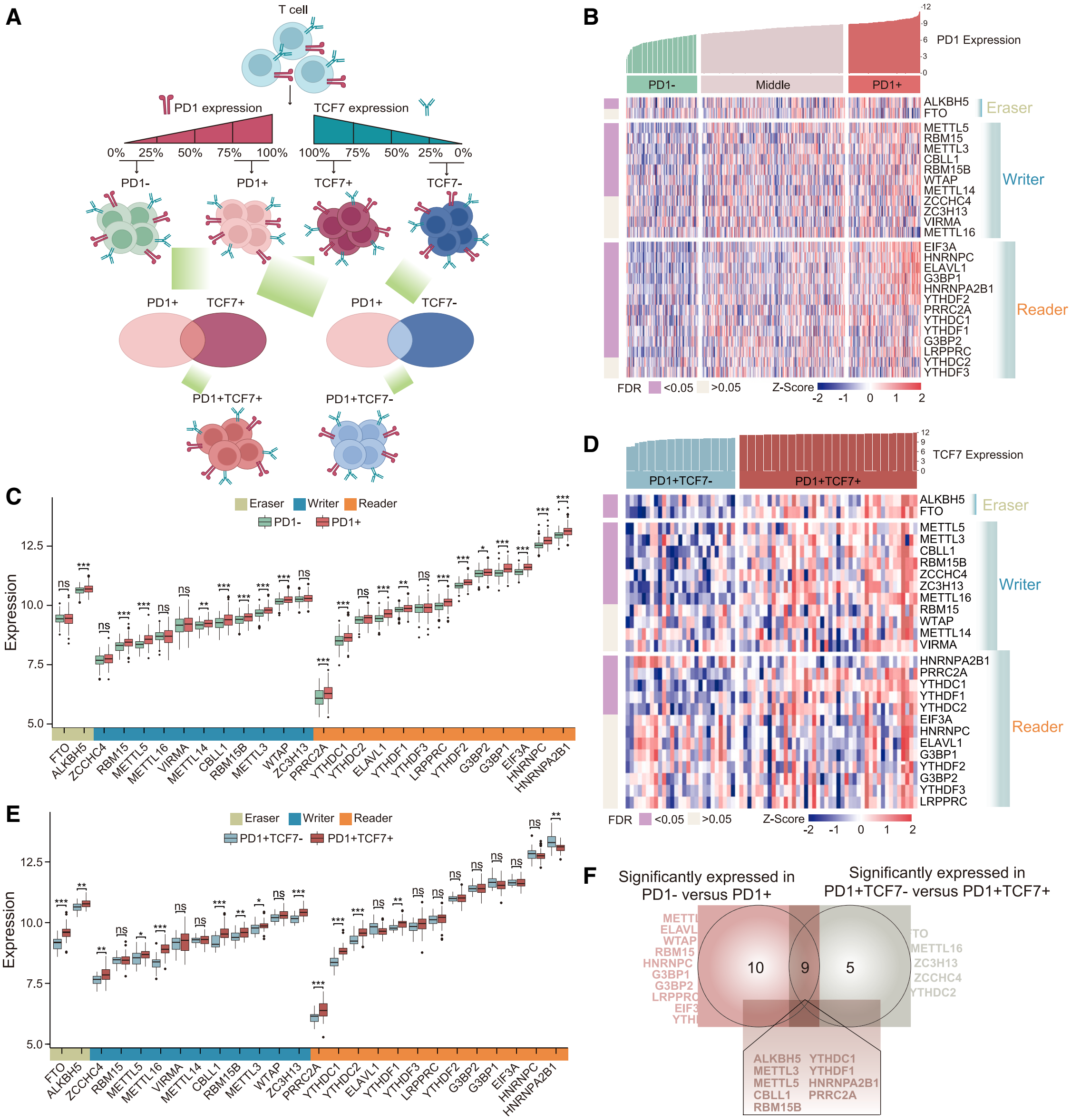

Figure 1. Expression pattern of m6A regulators during TEX in cell lines

(A) Flowchart of classifying cell line profile samples. (B) Expression heatmap for m6A genes in different PD1 expression groups. (C) Boxplots showing expression levels of m6A genes in different PD1 expression groups. (D) Expression heatmap for m6A genes in PD1^{+}TCF7^{-} versus PD1^{+}TCF7^{+} groups. (E) Boxplots showing expression levels of m6A genes in PD1^{+}TCF7^{-} versus PD1^{+}TCF7^{+} groups. (F) Venn diagram showing the numbers of significantly expressed genes between PD1^{-} versus PD1^{+} and PD1^{+}TCF7^{-} versus PD1^{+}TCF7^{+} groups.

comprises 11 writers, 2 erasers, and 18 readers, as detailed in Table S1. The up-regulation of checkpoint molecule PD-1 has been identified as a marker of an exhausted state of T cells. We classified 530 T cell line samples into states of exhaustion using PD-1 and TCF7 expression as primary markers. High PD-1 expression (75%–100%) was defined as an exhaustion state, while lower PD-1 levels (0%–25%) indicated a non-exhausted state. This led to the identification of progenitor TEX (PD1^{+}TCF7^{+}) and terminal TEX (PD1^{+}TCF7^{-}) groups. We first explored the involvement of m6A modifications in TEX by comparing the expression patterns of m6A regulators between T cell lines with high (top 25%) and low (bottom 25%) PD-1 expression (Figure 1A). Our analysis revealed 19 m6A regulators that exhibited statistically significant expression differences between normal (PD1^{-}) and exhausted (PD1^{+}) T cells (Figure 1B; Table S2). Specifically, the expression levels of the eraser ALKBH5, writers METTL5, RBM15, METTL3, CBLL1, RBM15B, WTAP, and METTL14, along with readers EIF3A, HNRNPC, ELAVL1, G3BP1, HNRNPA2B1, YTHDF2, PRRC2A, YTHDC1, YTHDF1, G3BP2,

and LRPPRC, were significantly elevated in exhausted T cells compared with normal T cells (Figure 1C). To assess the dynamic regulation of TEX by these 31 m6A regulators, we further examined their expression in progenitor Tex ($PD1^{+}TCF7^{+}$, 44 samples) and terminal Tex ($PD1^{+}TCF7^{-}$, 27 samples) subtypes (Figure 1A). Fourteen m6A regulators showed statistically significant expression differences between progenitor and terminal Tex (Figures 1D and 1E). Through cross-referencing, we discovered that 9 of the 31 m6A regulators were implicated in modulating TEX dynamics (Figure 1F).

Interaction between m6A regulators and TEX-related genes

To better understand the interaction between m6A regulators and TEX-related genes, we curated a list of 675 TEX-related genes and identified 305 of these genes that exhibit protein-protein interactions (PPIs) with m6A regulators (Figure 2A). Based on the T cell line dataset, we calculated the Pearson correlation coefficients between the 305 TEX-related genes and 26 m6A regulators. From this analysis, we identified 1,023 gene pairs with a significant correlation (adjusted $p < 0.05$ and $|r| > 0.2$), indicating a strong association between each gene pair (Figure 2A). By combining interaction and co-expression relationships, we constructed a functional interaction network comprising 204 gene nodes (26 m6A regulators and 178 TEX-related genes) (Figure 2B). Network analysis revealed that the majority of interactions between TEX-related genes and m6A regulators were positively regulated. Of the 439 gene pairs, 436 (99.32%) showed positive regulatory relationships, while only 3 pairs (0.68%) exhibited negative regulation. The negatively regulated pairs included ELAVL1-IFG1R, LRPPRC-RFHD2, and METT16-LDLR. Notably, ELAVL1 demonstrated extensive connectivity with TEX-related genes, showing the highest degree of association compared with other m6A regulators. Additionally, readers HNRNPA2B1, HNRNPC, and G3BP1 were also strongly linked with multiple TEX-related genes.

Single-cell expression of m6A regulators in TEX

We evaluated the expression patterns of 31 m6A regulators at the single-cell level using single-cell RNA sequencing (RNA-seq) data from $CD8^{+}$ T cells. Through dimensional reduction and clustering with the Seurat package, we visualized cell coordinates on a two-dimensional UMAP. The clustering analysis revealed 14 distinct cell clusters (Figure 2C). Using a set of known marker genes, we assigned cell types to each cluster, categorizing them into three primary cell types: cytotoxic T cells, exhausted T cells, and other T cells (Figure 2D). To investigate the involvement of m6A regulators in exhausted and cytotoxic T cells at the single-cell level, we compared the gene expression of 31 m6A regulators between these two cell types. Our analysis identified differential expression of 28 m6A regulators between cytotoxic and exhausted T cells (Table S3). Of these, 20 regulators were up-regulated in exhausted T cells, while 8 were downregulated) (Figure 2E). These findings are consistent with the expression trends observed at the cell line level, further confirming that the crosstalk between m6A modifications and TEX observed in cell lines is also evident at the single-cell level.

Crosstalk between m6A and TEX reveals de novel pan-cancer subtypes

Building on the observed interaction between m6A regulators and TEX-related genes, we performed an unsupervised consensus clustering analysis on the TCGA pan-cancer dataset, using the 204 genes (26 m6A regulators and 178 TEX-related genes) from the functional interaction network and identified three clusters designated as cluster A (n = 2,242), cluster B (n = 3,153), and cluster C (n = 4,092) (Figure S1). As expected, the three clusters demonstrate different expression levels of m6A regulators and varying levels of TEX, and we termed these clusters $Tex^{H}m6A^{H}$, $Tex^{L}m6A^{L}$, and $Tex^{L}m6A^{H}$ (Figure 3A). Kaplan-Meier survival analysis indicated significant survival differences among three subtypes, with improved survival in the $Tex^{L}m6A^{L}$ group and poor survival in the $Tex^{L}m6^{H}$ and $Tex^{H}m6^{H}$ groups (log rank $p < 0.001$) (Figure 3B).

We next assessed the distribution of three subtypes across 29 cancer types and observed varying subtype prevalence across different cancers. $Tex^{H}m6A^{H}$ was predominantly represented in cancers such as ACC, GBM, LGG, PRAD, and UCS, while $Tex^{L}m6A^{L}$ was more prevalent in cancers like BRCA, CHOL, KIRC, and SARC. $Tex^{L}m6A^{H}$ was largely distributed in cancers such as BLCA, CESC, LUAD, and UCEC (Figures 3D and 3E). Additionally, we explored the association between subtype distribution and clinical features, including gender, age, and cancer stage, and found significant differences among subtypes across these clinical characteristics (Figure 3F). Notably, female patients showed a higher prevalence of $Tex^{H}m6A^{H}$, whereas $Tex^{L}m6A^{L}$ was more common among males. Age distribution analysis revealed that $Tex^{L}m6A^{H}$ was least represented among younger patients (0–19 years) but increased with age, particularly among older adults ($\geq$60 years). Meanwhile, the prevalence of $Tex^{H}m6A^{H}$ decreased with age. Similarly, $Tex^{L}m6A^{H}$ prevalence increased with advancing cancer stages, whereas $Tex^{L}m6A^{L}$ showed a decline (Figure 3F). We conducted univariate and multivariate Cox regression analyses, adjusting for age, gender, and cancer stage, and revealed that the subtype classifications remained statistically significant for overall survival (Figure 3G).

Biological and immunological characterization of de novel pan-cancer subtypes

To explore the biological characteristics of the identified pan-cancer subtypes, we first identified genes specifically expressed in each subtype. A total of 170 genes were uniquely expressed in the $Tex^{H}m6A^{H}$, 88 in $Tex^{L}m6A^{L}$, and 499 in $Tex^{L}m6A^{H}$ (Figure 4A). Next, we performed Gene Ontology (GO) enrichment analysis for subtype-specific genes and found that $Tex^{H}m6A^{H}$-specific genes were significantly enriched in pathways related to developmental differentiation, particularly in the central nervous system and neuron differentiation (Figure 4B), $Tex^{L}m6A^{L}$ was primarily associated with metabolic and transport-related processes (Figure 4C) and $Tex^{L}m6A^{H}$ was enriched in development and defense mechanisms (Figure 4D). To further assess the functional differences between the subtypes, we evaluated the enrichment of hallmark gene sets from the MsigDB database. Our findings revealed distinct functional

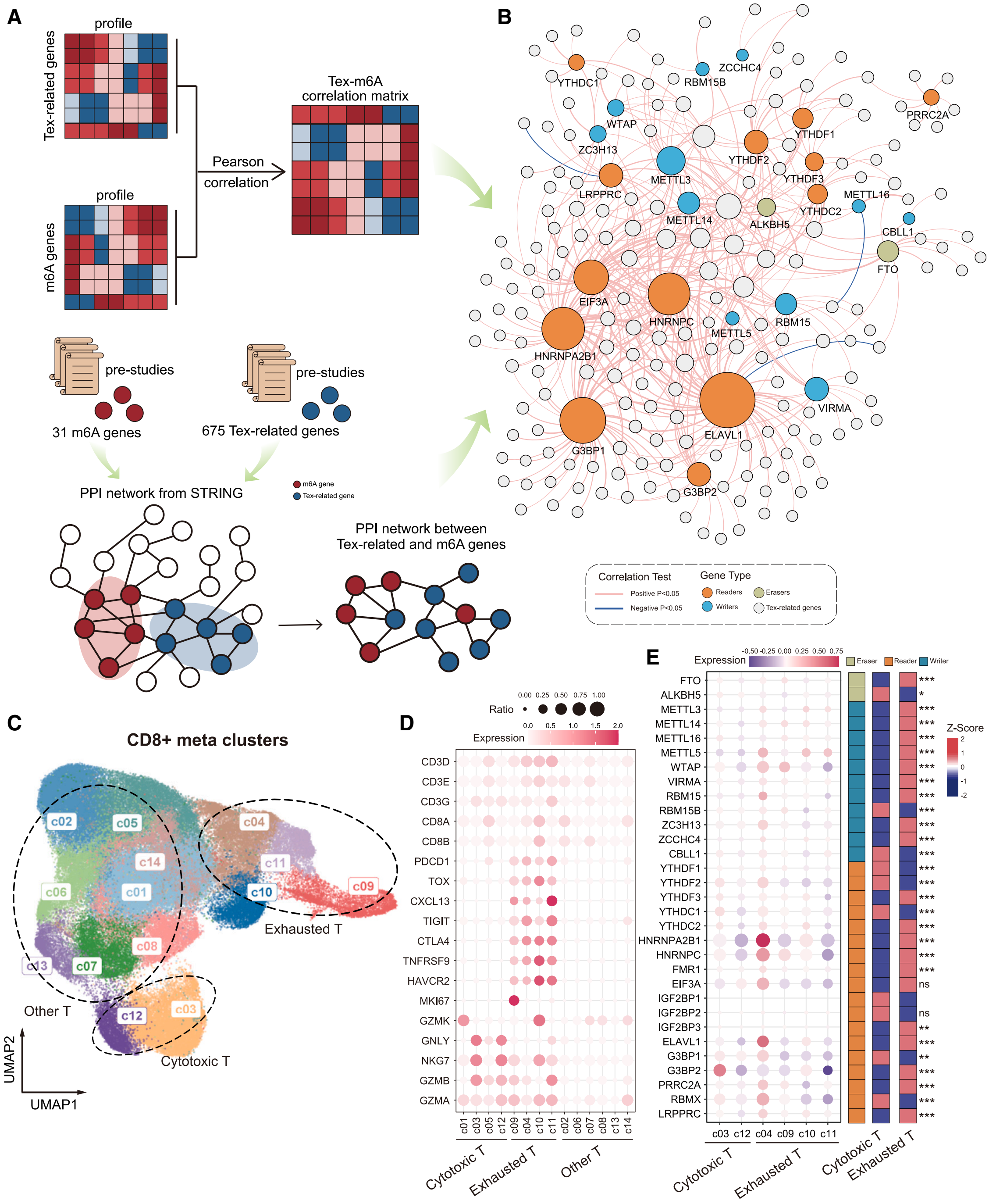

Figure 2. Crosstalk between m6A regulators and TEX genes at the single cell and network levels

(A) Flowchart of constructing functional association network between m6A regulators and TEX genes. (B) Functional association network between m6A regulators and TEX genes (C) UMAP visualization of $CD8^{+}$ T cell meta clusters. (D) Dot plot showing the expression of signature genes of the three $CD8^{+}$ T cells. Both color and size indicate the effect size. (E) Dot plot and heatmap showing expression of m6A regulators of Exhausted T and Cytotoxic T cells.

Selective metal clusters are highlighted by using their functional annotation: Cytotoxic T, cytotoxic T cells; Exhausted T, exhausted T cells; Other T, other T cells. ns, $p > 0.05$; *$p \leq 0.05$; **$p \leq 0.01$; ***$p \leq 0.001$.

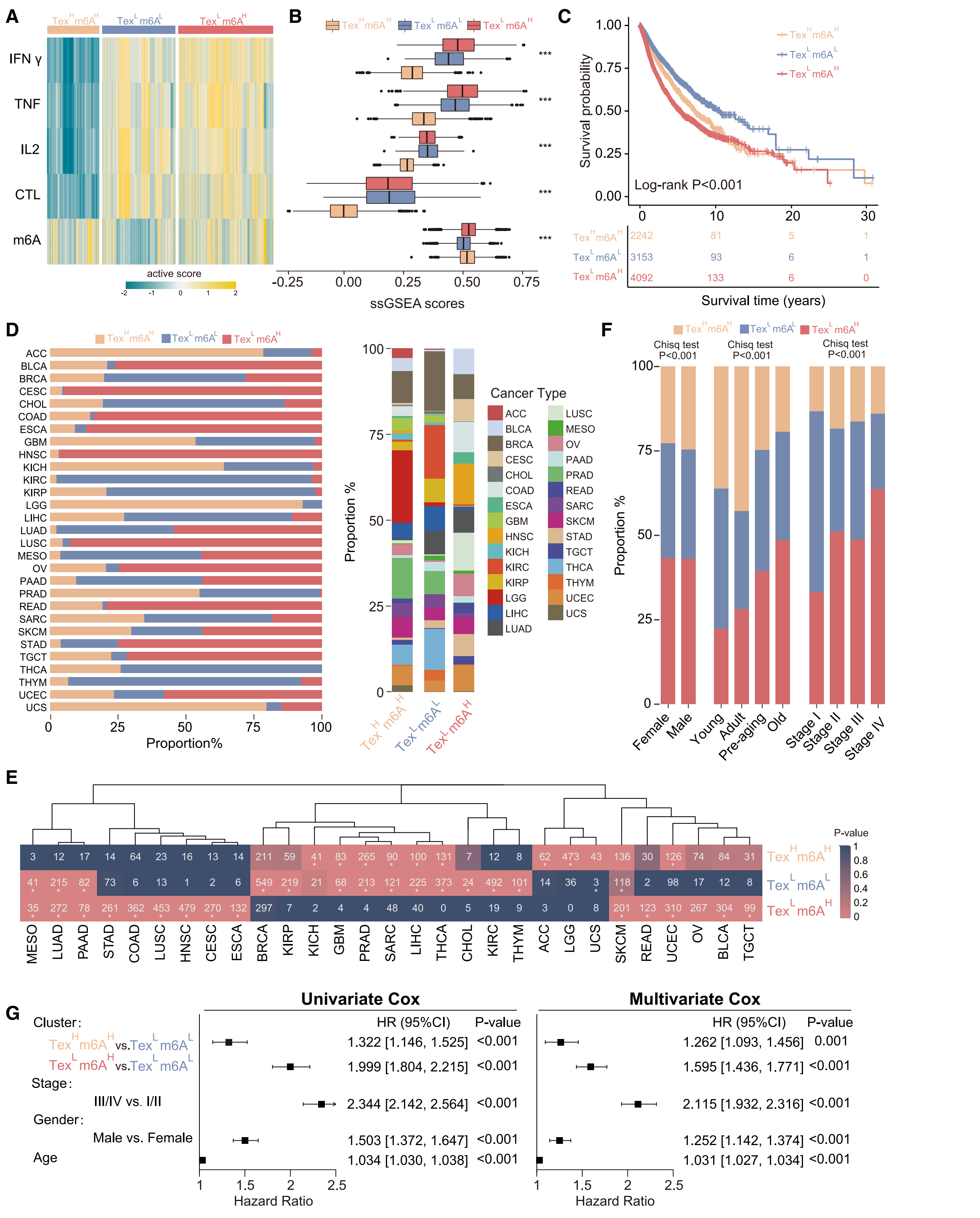

Figure 3. Identification of *de novo* pan-cancer subtypes based on m6A-TEX crosstalk

(A) Heatmap for active scores of four TEX-related pathways and m6A gene set in three pan-cancer subtypes. (B) Boxplots for active scores of four TEX-related pathways and m6A gene set in three pan-cancer subtypes. (C) Kaplan-Meier curves of overall survival among three pan-cancer subtypes. (D) Bar charts showing the distribution of the three

(legend continued on next page)

profiles for each subtype. $Tex^{L}m6A^{H}$ and $Tex^{H}m6A^{L}$ were both significantly enriched in immune, metabolic, and signaling pathways, while $Tex^{H}m6A^{H}$ showed a stronger association with pathways related to cell proliferation, such as MYC targets and G2/M checkpoints (Figures 4E and S2).

We further evaluated the TME features of the three subtypes by calculating activity scores for 29 functional gene expression signatures (Fges) covering known cellular and functional TME properties using the single sample gene set enrichment analysis (ssGSEA) (Figure 4F). These TME subtypes varied significantly based on the expression of the 29 Fges, and characterized by the presence of an immune-active or immunosuppressive microenvironment and tumor stroma (Figure 4F). The $Tex^{H}m6A^{H}$ subtype was characterized by minimal or absent immune cell infiltration, indicating an immune desert phenotype. The $Tex^{L}m6A^{H}$ subtype was characterized by the elevated expression of Fges associated with immune, angiogenesis, CAF activation, EMT transition, and cancer cell metastasis, while $Tex^{L}m6A^{L}$ were distinguished by high levels of immune infiltrate and significantly increased cytolytic score, suggesting a more immune-active microenvironment compared with the $Tex^{L}m6A^{H}$ subtype (Figure 4F).

Crosstalk between m6A modification and TEX predict the response of immunotherapy

To explore the relationships of m6A-TEX crosstalk with immunotherapy efficacy, we performed consensus clustering analysis for melanoma patients from independent immunotherapy cohorts. The clustering analysis revealed three distinct groups with significantly different survival outcomes and immune responses. The C3 cluster was associated with prolonged survival, while the C1 cluster exhibited poorer survival outcomes (Figures 5A and 5C). The percentage of responders to immunotherapy is significantly higher in patients in the C3 cluster compared with those in the C1 cluster. In the Gide cohort, 79.4% of patients in the C3 cluster responded to treatment, compared with only 35.7% in the C1 cluster. Similarly, in the IMvigor210 cohort, 38.5% of patients in the C3 cluster responded to immunotherapy, versus 15.0% in the C1 cluster (Figures 5B and 5D). These results indicate that the interplay between m6A modification and TEX could be an important predictor of patient response to immune checkpoint blockade therapy.

DISCUSSION

In this study, we conducted a comprehensive and pan-cancer analysis of the dynamic interplay between m6A RNA modification and TEX dynamics, providing valuable insights into the molecular mechanisms driving immune cell dysfunction in cancer and its implications for immunotherapy. By integrating multi-omics data from cell lines, single-cell RNA-seq, pan-cancer datasets, and immunotherapy cohorts, we identified three distinct pan-cancer subtypes based on m6A-TEX crosstalk, each with significant prognostic implications for patient survival and immunotherapy response.

Previous studies have shown that m6A modifications regulate T cell function and differentiation trajectories.[21–23] Our results corroborate these findings, demonstrating that m6A modification regulates the expression of key genes involved in TEX at both the cellular and single cell levels. By analyzing m6A regulators in a large cohort of cancer samples and immune cell datasets, we found that several m6A regulators are differentially expressed in exhausted T cells compared with normal T cells. Notably, we observed that YTHDF1, METTL3, and ALKBH5 are not only highly expressed in exhausted T cells but also progenitor TEX cells, consistent with previous findings.[24–26] Specifically, YTHDF1 and METTL3 have been reported to enhance myeloid-derived suppressor cell proliferation, which in turn inhibits $CD8^{+}$ T cell function across various cancer types. Additionally, METTL5 expression has been positively correlated with the degree of $CD8^{+}$ T cell infiltration in HCC.[27] Furthermore, we identified that m6A regulators such as HNRNPA2B1, ELAVL1, G3BP1, and HNRNPC are strongly associated with TEX. At the single-cell level, these regulators were highly expressed in exhausted T cells, with expression trends consistent with those observed at the cellular lineage level. A recent study also found that the knockdown of HNRNPC was associated with increased $CD8^{+}$ T cell infiltration.[28]

Our study also extends previous work on TEX subtypes by integrating m6A regulation.[10,29,30] Through unsupervised clustering of pan-cancer datasets, we identified three novel subtypes based on m6A-TEX crosstalk: $Tex^{H}m6A^{H}$, $Tex^{L}m6A^{L}$, and $Tex^{L}m6A^{H}$. These subtypes were associated with distinct functional characteristics and immune phenotypes, providing a novel framework for understanding the heterogeneity of TEX in cancer and its regulation by m6A modifications. The $Tex^{L}m6A^{L}$ subtype, characterized by low m6A activity and low TEX, was associated with favorable immune responses and better survival outcomes in several cancer types. In contrast, the $Tex^{H}m6A^{H}$ subtype, with high m6A activity and high exhaustion, was linked to poorer clinical outcomes and immune evasion. Furthermore, the association between m6A-TEX subtypes and immune therapy response in two independent immunotherapy cohorts (IMvigor210 and Gide) provides compelling evidence that m6A modifications can predict patient responses to ICB. The $Tex^{L}m6A^{L}$ subtype showed enhanced clinical benefit from immunotherapy, while the $Tex^{H}m6A^{H}$ subtype demonstrated reduced treatment efficacy. These findings suggest that manipulating m6A pathways to restore T cell function could represent a promising therapeutic strategy to improve ICB outcomes in cancer patients.[24,31]

However, several limitations in this study should be noted. First, our study provides only bioinformatic evidence for the role of m6A

pan-cancer subtypes among the different tumor types. (E) Bar charts showing the distribution of the three pan-cancer subtypes among the different clinical features. (F) Number of cases in each pan-cancer subtype across different cancer types. Colors represent the *p*-value calculated from a hypergeometric test comparing the fraction of samples of a given cancer type in a subtype to the fraction of samples that are in that subtype overall. (G) Forest plots for univariate and multivariate Cox regression analysis.

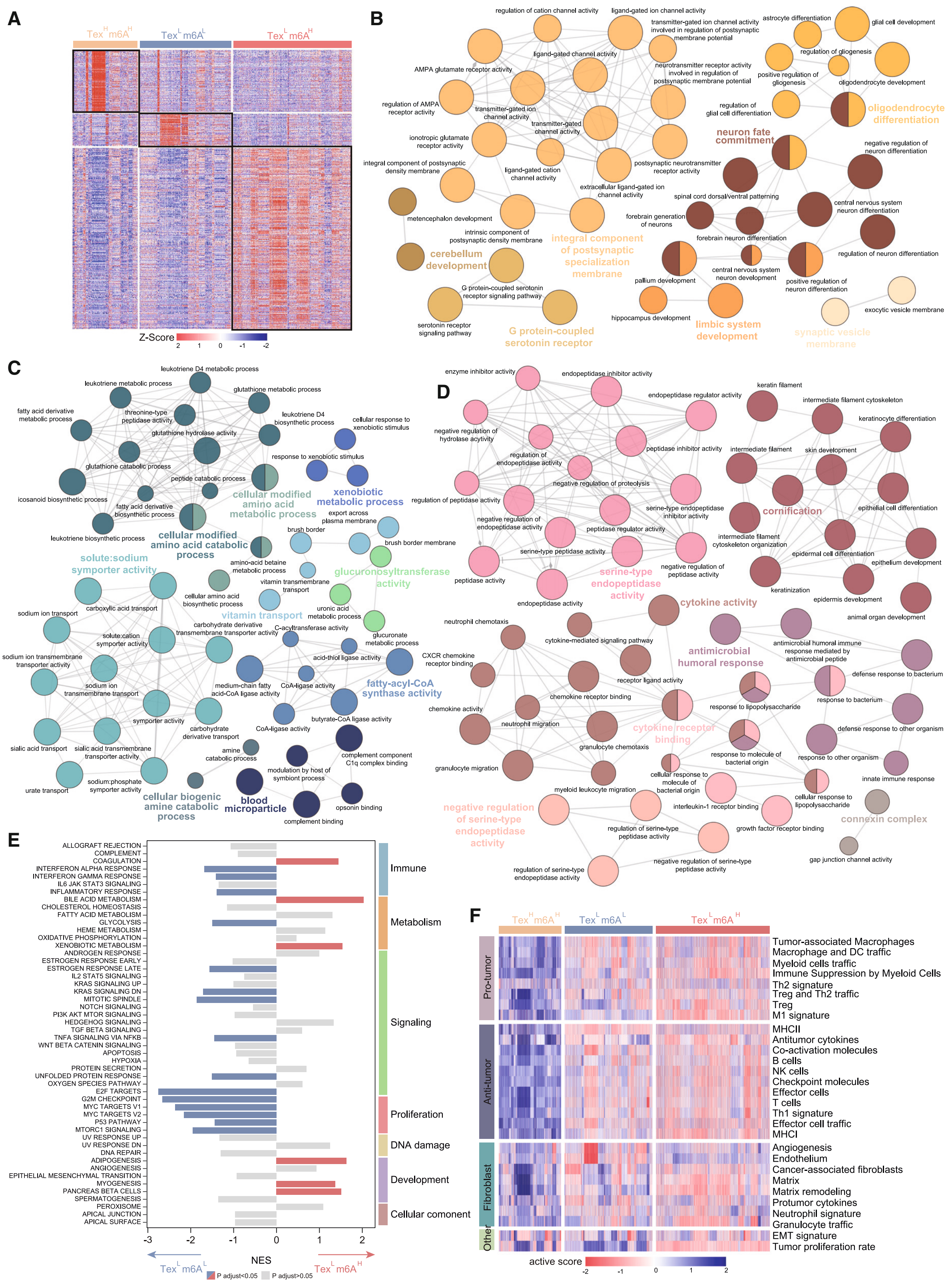

(legend on next page)

regulators in TEX, experimental validation is needed to confirm these findings. Second, while our study focuses on $CD8^+$ T cells, TEX also affects other immune cell populations, including $CD4^+$ T cells. Finally, although we assessed the clinical relevance of m6A-TEX crosstalk using immunotherapy cohorts, larger clinical trials are necessary to validate these findings in real-world settings.

In conclusion, our study provides a comprehensive analysis of the regulatory role of m6A modification in TEX dynamics across multiple cancer types. By identifying novel m6A-TEX subtypes and exploring their biological and immunological features, our study not only expands the understanding of TEX dynamics but also highlights the potential of m6A regulators as biomarkers and therapeutic targets to improve cancer immunotherapy.

MATERIALS AND METHODS

Pan-cancer multi-omics dataset

Pan-cancer multi-omics data and clinical information of 29 solid tumor types (ACC, BLCA, BRCA, CESC, CHOL, COAD, ESCA, GBM, HNSC, KICH, KIRC, KIRP, LGG, LIHC, LUAD, LUSC, MESO, OV, PAAD, PRAD, READ, SARC, SKCM, STAD, TGCT, THCA, THYM, UCEC, and UCS) were obtained from The Cancer Genome Atlas (TCGA) through the UCSC Xena (https://xena.ucsc.edu/). Samples lacking survival information or originating from the TARGET project were excluded. A total of 9,487 samples were included in this study.

Single-cell RNA-seq dataset

Pan-cancer single-cell RNA-seq data of tumor-infiltrating T cells was obtained from the Gene Expression Omnibus (GEO) under the access number GSE156728 (https://www.ncbi.nlm.nih.gov/geo/query/acc.cgi?acc=GSE156728).[32] The dataset includes $CD8^+$ T cells from eight cancer types: BC (breast cancer), BCL (B-cell lymphoma), ESCA (esophageal cancer), MM (multiple myeloma), PACA (pancreatic cancer), RC (renal carcinoma), THCA (thyroid carcinoma), and UCEC (uterine corpus endometrial carcinoma). Dimensionality reduction and clustering of the single-cell RNA-seq data were performed using Seurat (version 4.3.0).

Cell line dataset

Cell line-normalized RNA-seq data based on the Illumina HiSeq platform were obtained for 28 immune cell subsets from the National Bioscience Database Center Human Database (https://humandbs.biosciencedbc.jp/en/) with the accession number of E-GEAD-397. We integrated EM and TEMRA T cell lines using the sva R package (version 3.42.0).

Immunotherapy dataset

Two immunotherapy cohorts were used in our study, including 61 patients with metastatic urothelial cancer who were treated with an anti-PD-L1 agent (IMvigor210 cohort) from Mariathasan et al.'s study,[33] and 91 melanoma patients treated with anti-PD-1 monotherapy or combined anti-PD-1 and anti-CTLA-4 (Gide cohort) from Gide et al.'s study.[34] The FASTQ files from both cohorts were quality controlled and adapter trimmed using Trim Galore. The reads were then quantified using Homo_gencodeV29_transcripts.idx as a reference with kallisto.[35] The transcript-level expression data were converted to gene-level data using the transcript2gene function from the BUSpaRse R package.

Curation of TEX and m6A modification-related gene sets

We curated a comprehensive set of genes related to TEX and m6A modification by reviewing publicly available literature. A total of 518 TEX-related genes and 31 m6A regulators were identified and compiled for further analysis (Table S1).

Construction of a functional association network

The global human PPI data were derived from the STRING database (version 11.5) (https://string-db.org/).[36] Gene and protein mapping was performed using annotation files from GENCODE (version 38).[37] Gene-gene co-expression associations were determined using Pearson correlation coefficients, selecting gene pairs with adjusted $p < 0.05$ and $|r| > 0.2$. The final functional association network was created by integrating both PPI and gene co-expression data.

Enrichment analysis

ssGSEA for 29 Fges from Newel et al.'s study[38] was performed using the GSVA R package (version 1.42.0).[39] GO and Kyoto Encyclopedia of Genes and Genomes pathway enrichment analyses were conducted using ClueGO in Cytoscape (version 3.9.0).[40] GSEA for hallmarker gene sets from the Molecular Signatures Database (http://software.broad-institute.org/gsea/msigdb) was performed using the clusterProfiler R package (version 4.2.2).[41]

Unsupervised clustering

Unsupervised clustering analysis was conducted using the ConsensuClusterPlus R package (version 1.58.0) with 1,000 repetitions, K-means algorithm, and Pearson correlation as the distance measure. Cluster stability and the optimal number of clusters were evaluated based on item consensus and cluster consensus.

Differential gene expression analysis

Differential gene expression analysis was performed using the limma R package with empirical Bayesian statistics.[42] Genes with a false discovery rate of <0.01 and |logFC| >2 were considered differentially expressed genes.

Statistical analyses

All statistical analyses and data visualization were conducted in R (version 4.1.0). Correlations coefficients between two continuous variables were computed by Spearman and distance correlation

Figure 4. Biological and immunological characterization of de novel pan-cancer subtype

(A) Expression heatmap of subtype-specific genes. GO enrichment analysis of subtype-specific genes for $Tex^H m6A^H$ (B), $Tex^L m6A^L$, (C) and $Tex^L m6A^H$ (D). (E) Enrichment analysis for the MSigDB hallmark pathways. (F) Heatmap for the 29 Fges of three pan-cancer subtypes.

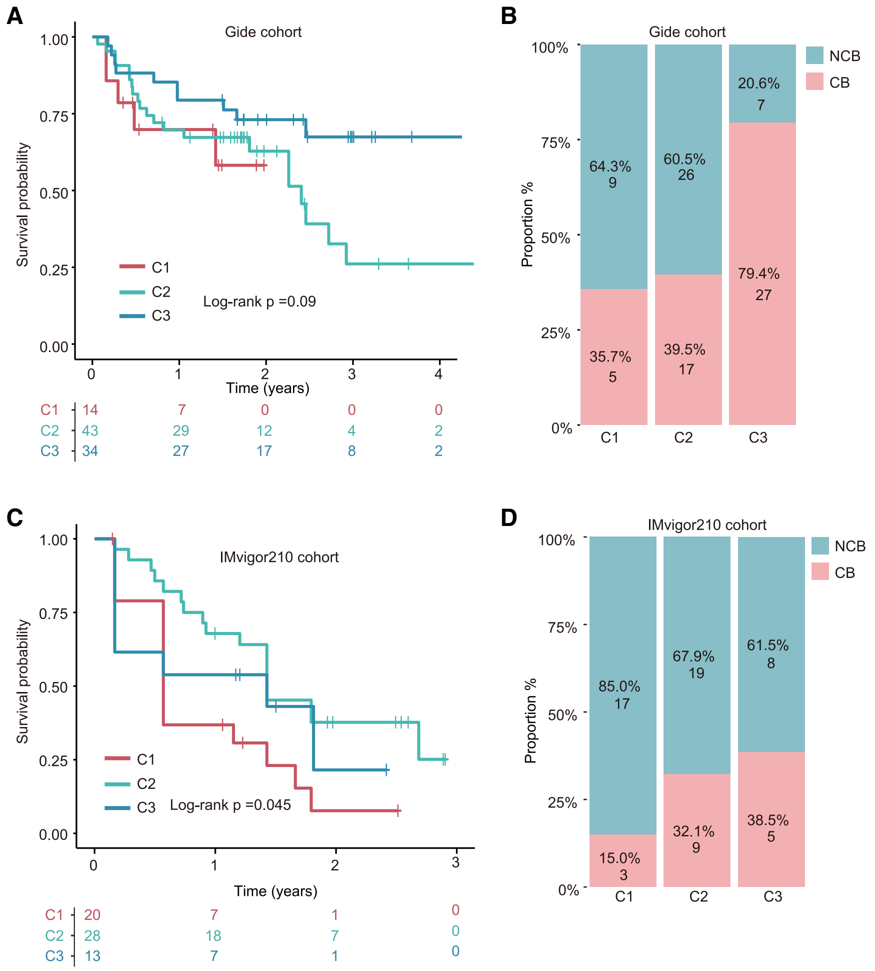

Figure 5. Correlation of m6A-TEX crosstalk with immunotherapy response

Kaplan-Meier curves of overall survival among three groups in the Gide cohort (A) and IMvigor210 cohort (C). The proportion of responders in each patient group in the Gide cohort (B) and IMvigor210 cohort (D).

analyses. The unpaired Student's t test was used for normally distributed variables, and the Wilcoxon test for non-normally distributed variables. The hypergeometric test was used to assess sample bias distribution among clusters. Kaplan-Meier survival curves were generated and compared using log rank tests. Univariate and multivariate Cox regression models were used to calculate hazard ratios for clinical variables, with results visualized using the forestplot R package (version 3.1.1). A *p* value of <0.05 was considered statistically significant for all analyses.

DATA AND CODE AVAILABILITY

Pan-cancer multi-omics data and clinical information of 29 solid tumor types were obtained from TCGA through the UCSC Xena (https://xena.ucsc.edu/). Pan-cancer single-cell RNA-seq data of tumor-infiltrating T cells was obtained from the GEO under the access number GSE156728 (https://www.ncbi.nlm.nih.gov/geo/query/acc.cgi?acc=GSE156728). All source code for data analysis is currently hosted on GitHub (https://github.com/ZhoulabCPH/TEX-m6A.git).

ACKNOWLEDGMENTS

This study was supported by the Natural Science Foundation of Zhejiang Province (No. LTGY23H030003). The funders had no roles in study design, data collection and analysis, publication decision, or manuscript preparation.

AUTHOR CONTRIBUTIONS

M.Z. contributed to conception and design; W.P.J., Y.F., L.W.C., Y.T.Z., Y.F.P., and C.Q.M. contributed to data analysis and interpretation. W.P.J. and Y.F. drafted the manuscript. All authors read and approved the final manuscript.

DECLARATION OF INTERESTS

The authors declare no conflict of interest.

SUPPLEMENTAL INFORMATION

Supplemental information can be found online at https://doi.org/10.1016/j.omtn.2025.102465.

REFERENCES

1. Wherry, E.J. (2011). T cell exhaustion. Nat. Immunol. *12*, 492–499. https://doi.org/10.1038/ni.2035.

2. Blank, C.U., Haining, W.N., Held, W., Hogan, P.G., Kallies, A., Lugli, E., Lynn, R.C., Philip, M., Rao, A., Restifo, N.P., et al. (2019). Defining 'T cell exhaustion. Nat. Rev. Immunol. *19*, 665–674. https://doi.org/10.1038/s41577-019-0221-9.

3. Wherry, E.J., and Kurachi, M. (2015). Molecular and cellular insights into T cell exhaustion. Nat. Rev. Immunol. *15*, 486–499. https://doi.org/10.1038/nri3862.

4. Zebley, C.C., and Youngblood, B. (2022). Mechanisms of T cell exhaustion guiding next-generation immunotherapy. Trends Cancer *8*, 726–734. https://doi.org/10.1016/j.trecan.2022.04.004.

5. Barsch, M., Salié, H., Schlaak, A.E., Zhang, Z., Hess, M., Mayer, L.S., Tauber, C., Otto-Mora, P., Ohtani, T., Nilsson, T., et al. (2022). T-cell exhaustion and residency dynamics inform clinical outcomes in hepatocellular carcinoma. J. Hepatol. *77*, 397–409. https://doi.org/10.1016/j.jhep.2022.02.032.

6. Beltra, J.C., Manne, S., Abdel-Hakeem, M.S., Kurachi, M., Giles, J.R., Chen, Z., Casella, V., Ngiow, S.F., Khan, O., Huang, Y.J., et al. (2020). Developmental Relationships of Four Exhausted CD8(+) T Cell Subsets Reveals Underlying Transcriptional and Epigenetic Landscape Control Mechanisms. Immunity *52*, 825–841.e8. https://doi.org/10.1016/j.immuni.2020.04.014.

7. Crespo, J., Sun, H., Welling, T.H., Tian, Z., and Zou, W. (2013). T cell anergy, exhaustion, senescence, and stemness in the tumor microenvironment. Curr. Opin. Immunol. *25*, 214–221. https://doi.org/10.1016/j.coi.2012.12.003.

8. Jiang, Y., Li, Y., and Zhu, B. (2015). T-cell exhaustion in the tumor microenvironment. Cell Death Dis. *6*, e1792. https://doi.org/10.1038/cddis.2015.162.

9. Tille, L., Cropp, D., Charmoy, M., Reichenbach, P., Andreatta, M., Wyss, T., Bodley, G., Crespo, I., Nassiri, S., Lourenco, J., et al. (2023). Activation of the transcription factor NFAT5 in the tumor microenvironment enforces CD8(+) T cell exhaustion. Nat. Immunol. *24*, 1645–1653. https://doi.org/10.1038/s41590-023-01614-x.

10. Zhang, Z., Chen, L., Chen, H., Zhao, J., Li, K., Sun, J., and Zhou, M. (2022). Pan-cancer landscape of T-cell exhaustion heterogeneity within the tumor microenvironment revealed a progressive roadmap of hierarchical dysfunction associated with prognosis and therapeutic efficacy. EBioMedicine *83*, 104207. https://doi.org/10.1016/j.ebiom.2022.104207.

11. Yan, D., Bao, S., Zhang, Z., Sun, J., and Zhou, M. (2024). Leveraging pharmacovigilance data to predict population-scale toxicity profiles of checkpoint inhibitor immunotherapy. Nat. Comput. Sci. https://doi.org/10.1038/s43588-024-00748-8.

12. Roost, C., Lynch, S.R., Batista, P.J., Qu, K., Chang, H.Y., and Kool, E.T. (2015). Structure and thermodynamics of N6-methyladenosine in RNA: a spring-loaded base modification. J. Am. Chem. Soc. *137*, 2107–2115. https://doi.org/10.1021/ja513080v.

13. Hong, J., Xu, K., and Lee, J.H. (2022). Biological roles of the RNA m(6)A modification and its implications in cancer. Exp. Mol. Med. *54*, 1822–1832. https://doi.org/10.1038/s12276-022-00897-8.

14. Jiang, X., Liu, B., Nie, Z., Duan, L., Xiong, Q., Jin, Z., Yang, C., and Chen, Y. (2021). The role of m6A modification in the biological functions and diseases. Signal Transduct. Targeted Ther. *6*, 74. https://doi.org/10.1038/s41392-020-00450-x.

15. Zha, L.F., Wang, J.L., and Cheng, X. (2022). The effects of RNA methylation on immune cells development and function. FASEB J. *36*, e22552. https://doi.org/10.1096/fj.202200716R.

16. Zhang, Y., Hu, W., and Li, H.B. (2023). RNA modification-mediated translational control in immune cells. RNA Biol. *20*, 603–613. https://doi.org/10.1080/15476286.2023.2246256.

17. Lou, X., Wang, J.J., Wei, Y.Q., and Sun, J.J. (2021). Emerging role of RNA modification N6-methyladenosine in immune evasion. Cell Death Dis. *12*, 300. https://doi.org/10.1038/s41419-021-03585-z.

18. Cao, X., Geng, Q., Fan, D., Wang, Q., Wang, X., Zhang, M., Zhao, L., Jiao, Y., Deng, T., Liu, H., et al. (2023). m(6)A methylation: a process reshaping the tumour immune microenvironment and regulating immune evasion. Mol. Cancer *22*, 42. https://doi.org/10.1186/s12943-022-01704-8.

19. Zhang, N., Yang, F., Zhao, P., Jin, N., Wu, H., Liu, T., Geng, Q., Yang, X., and Cheng, L. (2023). MrGPS: an m6A-related gene pair signature to predict the prognosis and immunological impact of glioma patients. Briefings Bioinf. *25*, bbad498. https://doi.org/10.1093/bib/bbad498.

20. Zu, H., and Chen, X. (2024). Epigenetics behind CD8(+) T cell activation and exhaustion. Gene Immun. *25*, 525–540. https://doi.org/10.1038/s41435-024-00307-1.

21. Dong, L., Chen, C., Zhang, Y., Guo, P., Wang, Z., Li, J., Liu, Y., Liu, J., Chang, R., Li, Y., et al. (2021). The loss of RNA N(6)-adenosine methyltransferase Mettl14 in tumor-associated macrophages promotes CD8(+) T cell dysfunction and tumor growth. Cancer Cell *39*, 945–957.e10. https://doi.org/10.1016/j.ccell.2021.04.016.

22. Elsabbagh, R.A., Rady, M., Watzl, C., Abou-Aisha, K., and Gad, M.Z. (2022). Impact of N6-methyladenosine (m(6)A) modification on immunity. Cell Commun. Signal. *20*, 140. https://doi.org/10.1186/s12964-022-00939-8.

23. Li, H.B., Tong, J., Zhu, S., Batista, P.J., Duffy, E.E., Zhao, J., Bailis, W., Cao, G., Kroehling, L., Chen, Y., et al. (2017). m(6)A mRNA methylation controls T cell homeostasis by targeting the IL-7/STAT5/SOCS pathways. Nature *548*, 338–342. https://doi.org/10.1038/nature23450.

24. Bao, Y., Zhai, J., Chen, H., Wong, C.C., Liang, C., Ding, Y., Huang, D., Gou, H., Chen, D., Pan, Y., et al. (2023). Targeting m(6)A reader YTHDF1 augments antitumour immunity and boosts anti-PD-1 efficacy in colorectal cancer. Gut *72*, 1497–1509. https://doi.org/10.1136/gutjnl-2022-328845.

25. Chen, H., Pan, Y., Zhou, Q., Liang, C., Wong, C.C., Zhou, Y., Huang, D., Liu, W., Zhai, J., Gou, H., et al. (2022). METTL3 Inhibits Antitumor Immunity by Targeting m(6)A-BHLHE41-CXCL1/CXCR2 Axis to Promote Colorectal Cancer. Gastroenterology *163*, 891–907. https://doi.org/10.1053/j.gastro.2022.06.024.

26. Wang, L., Zhu, L., Liang, C., Huang, X., Liu, Z., Huo, J., Zhang, Y., Zhang, Y., Chen, L., Xu, H., et al. (2023). Targeting N6-methyladenosine reader YTHDF1 with siRNA boosts antitumor immunity in NASH-HCC by inhibiting EZH2-IL-6 axis. J. Hepatol. *79*, 1185–1200. https://doi.org/10.1016/j.jhep.2023.06.021.

27. Wang, L., and Peng, J.L. (2023). METTL5 serves as a diagnostic and prognostic biomarker in hepatocellular carcinoma by influencing the immune microenvironment. Sci. Rep. *13*, 10755. https://doi.org/10.1038/s41598-023-37807-5.

28. Gu, Z., Yang, Y., Ma, Q., Wang, H., Zhao, S., Qi, Y., and Li, Y. (2022). HNRNPC, a predictor of prognosis and immunotherapy response based on bioinformatics analysis, is related to proliferation and invasion of NSCLC cells. Respir. Res. *23*, 362. https://doi.org/10.1186/s12931-022-02227-y.

29. Zhang, C., Sheng, Q., Zhang, X., Xu, K., Jin, X., Zhou, W., Zhang, M., Lv, D., Yang, C., Li, Y., et al. (2023). Prioritizing exhausted T cell marker genes highlights immune subtypes in pan-cancer. iScience *26*, 106484. https://doi.org/10.1016/j.isci.2023.106484.

30. Zhong, J., Liu, Z., Cai, C., Duan, X., Deng, T., and Zeng, G. (2021). m(6)A modification patterns and tumor immune landscape in clear cell renal carcinoma. J. Immunother. Cancer *9*, e001646. https://doi.org/10.1136/jitc-2020-001646.

31. Li, X., Ma, S., Deng, Y., Yi, P., and Yu, J. (2022). Targeting the RNA m(6)A modification for cancer immunotherapy. Mol. Cancer *21*, 76. https://doi.org/10.1186/s12943-022-01558-0.

32. Zheng, L., Qin, S., Si, W., Wang, A., Xing, B., Gao, R., Ren, X., Wang, L., Wu, X., Zhang, J., et al. (2021). Pan-cancer single-cell landscape of tumor-infiltrating T cells. Science *374*, abe6474. https://doi.org/10.1126/science.abe6474.

33. Mariathasan, S., Turley, S.J., Nickles, D., Castiglioni, A., Yuen, K., Wang, Y., Kadel, E.E., III, Koeppen, H., Astarita, J.L., Cubas, R., et al. (2018). TGFbeta attenuates tumour response to PD-L1 blockade by contributing to exclusion of T cells. Nature *554*, 544–548. https://doi.org/10.1038/nature25501.

34. Gide, T.N., Quek, C., Menzies, A.M., Tasker, A.T., Shang, P., Holst, J., Madore, J., Lim, S.Y., Velickovic, R., Wongchenko, M., et al. (2019). Distinct Immune Cell Populations Define Response to Anti-PD-1 Monotherapy and Anti-PD-1/Anti-CTLA-4 Combined Therapy. Cancer Cell *35*, 238–255.e6. https://doi.org/10.1016/j.ccell.2019.01.003.

35. Bray, N.L., Pimentel, H., Melsted, P., and Pachter, L. (2016). Near-optimal probabilistic RNA-seq quantification. Nat. Biotechnol. *34*, 525–527. https://doi.org/10.1038/nbt.3519.

36. Szklarczyk, D., Gable, A.L., Lyon, D., Junge, A., Wyder, S., Huerta-Cepas, J., Simonovic, M., Doncheva, N.T., Morris, J.H., Bork, P., et al. (2019). STRING v11: protein-protein association networks with increased coverage, supporting functional discovery in genome-wide experimental datasets. Nucleic Acids Res. *47*, D607–D613. https://doi.org/10.1093/nar/gky1131.

37. Frankish, A., Diekhans, M., Jungreis, I., Lagarde, J., Loveland, J.E., Mudge, J.M., Sisu, C., Wright, J.C., Armstrong, J., Barnes, I., et al. (2021). Gencode 2021. Nucleic Acids Res. *49*, D916–D923. https://doi.org/10.1093/nar/gkaa1087.

38. Newell, F., Pires da Silva, I., Johansson, P.A., Menzies, A.M., Wilmott, J.S., Addala, V., Carlino, M.S., Rizos, H., Nones, K., Edwards, J.J., et al. (2022). Multiomic profiling of checkpoint inhibitor-treated melanoma: Identifying predictors of response and resistance, and markers of biological discordance. Cancer Cell *40*, 88–102.e7. https://doi.org/10.1016/j.ccell.2021.11.012.

39. Hanzelmann, S., Castelo, R., and Guinney, J. (2013). GSVA: gene set variation analysis for microarray and RNA-seq data. BMC Bioinf. *14*, 7. https://doi.org/10.1186/1471-2105-14-7.

40. Bindea, G., Mlecnik, B., Hackl, H., Charoentong, P., Tosolini, M., Kirilovsky, A., Fridman, W.H., Pagès, F., Trajanoski, Z., and Galon, J. (2009). ClueGO: a Cytoscape plug-in to decipher functionally grouped gene ontology and pathway annotation networks. Bioinformatics *25*, 1091–1093. https://doi.org/10.1093/bioinformatics/btp101.

41. Yu, G., Wang, L.G., Han, Y., and He, Q.Y. (2012). clusterProfiler: an R package for comparing biological themes among gene clusters. OMICS *16*, 284–287. https://doi.org/10.1089/omi.2011.0118.

42. Ritchie, M.E., Phipson, B., Wu, D., Hu, Y., Law, C.W., Shi, W., and Smyth, G.K. (2015). limma powers differential expression analyses for RNA-sequencing and microarray studies. Nucleic Acids Res. *43*, e47. https://doi.org/10.1093/nar/gkv007.

OMTN, Volume *36*

Supplemental information

Pan-cancer characterization of m6A-mediated regulation of T cell exhaustion dynamics and clinical relevancies in human cancers

Weiping Ji, Ye Fang, Liwei Chen, Yitong Zheng, Yifei Pei, Changqiu Mei, and Meng Zhou

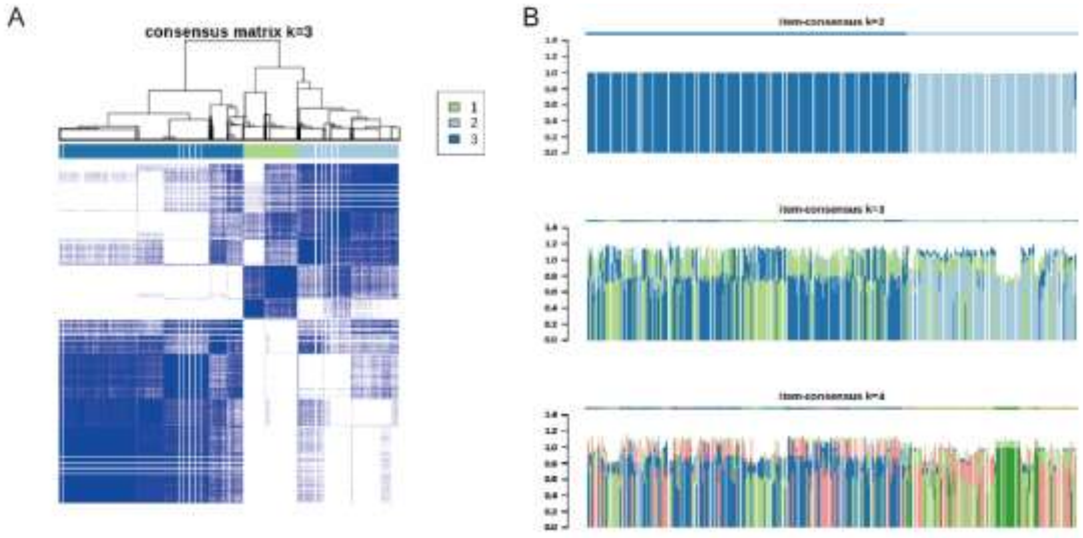

Figure S1. Consensus clustering. (A) Consensus matrix heatmap of the chosen optimal cluster number (k =3). (B) Item consensus for different cluster number

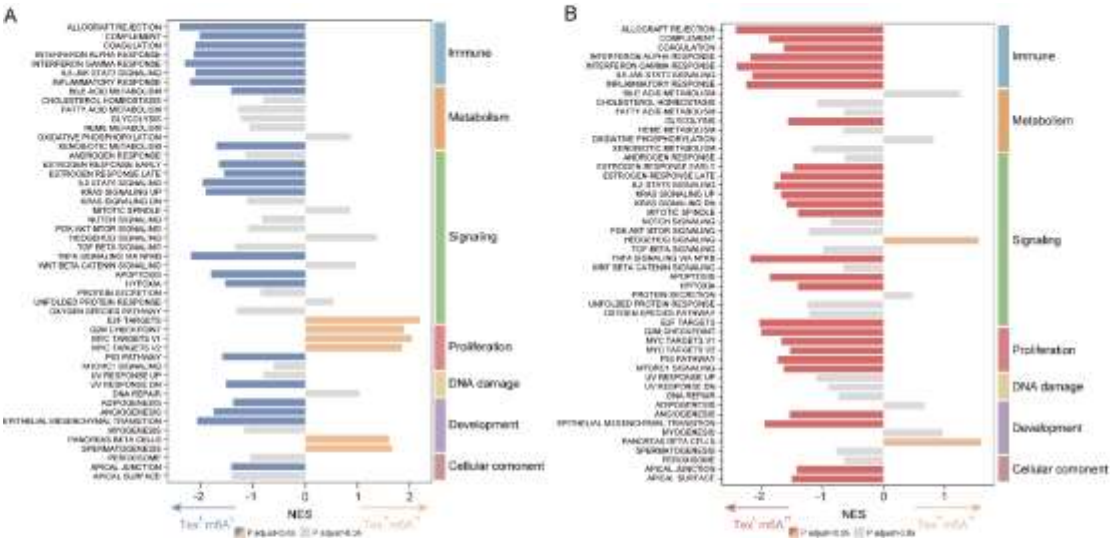

Figure S2. Enrichment analysis for the MSigDB hallmark pathways between $\text{TexLm6A}^{\text{L}}$ and $\text{TexHm6A}^{\text{H}}$ (A), and between $\text{Tex}^{\text{L}}\text{m6A}^{\text{H}}$ and $\text{Tex}^{\text{H}}\text{m6A}^{\text{H}}$ (B).

Supplemental Tables

Table S1. List of 518 T-cell exhaustion related genes and 31 m6A regulators.

Table S2. List of differentially expressed m6A regulators between normal T cells and exhausted T cells,

Table S3. List of differentially expressed m6A regulators between cytotoxic and exhausted T cells.
